# Targeting motifs in frustule-associated proteins from the centric diatom *Thalassiosira pseudonana*

**DOI:** 10.3389/fpls.2022.1006072

**Published:** 2022-10-28

**Authors:** Neri Fattorini, Uwe G. Maier

**Affiliations:** ^1^ Department of Biology, Laboratory for Cell Biology Philipps-University Marburg, Marburg, Germany; ^2^ Center for Synthetic Microbiology (SYNMIKRO), Philipps-University Marburg, Marburg, Germany

**Keywords:** silaffins, cingulins, frustule, silica, pentalysine clusters, lysine-enriched regions

## Abstract

The frustule of diatoms has an exceptional structure composed of inorganic and organic molecules. In the organic fraction, protein families were identified whose members are expected to have a complex cellular targeting to their final location within the frustule. Here we investigated for frustule-targeting signals two representatives of the cingulin family, the proteins CinY2 and CinW2; beside an already known, classical signal peptide, we have identified further regions involved in cellular targeting. By using these regions as a search criteria we were able to identify two new frustule proteins. In addition, we showed that the temporal regulation of the gene expression determines the final location of one cingulin. Our results therefore point to a sophisticated cellular and extracellular targeting of frustule components to build the fascinating frustule structure of a diatom.

## Introduction

Diatoms are unicellular photosynthetic eukaryotes (Graham et al., 2016) with a huge impact on the global scale in respect to oxygenic photosynthesis (Armbrust, 2009), primary production (Field et al., 1998), carbon (Tréguer and Pondaven, 2000) and silicon (Tréguer and De La Rocha, 2013) cycling. These organisms represent one of the most important (Falkowski et al., 2004) and diversified phytoplankton taxa, with 200000 estimated species (Mann and Droop, 1996). Diatoms are ubiquitous in the world’s aquatic environment, frequently dominating the phytoplankton, especially when nutrients and light are abundant (Armbrust, 2009). For centuries, diatoms have fascinated scientists and amateur naturalists with their beautifully decorated silica-based cell wall, the frustule. This complex biological structure is a wonderful example of biomineralization, which is the process through which organisms can form solid inorganic structures using the resources available from the natural environment (Richthammer et al., 2011). Diatoms, in fact, synthesize their silica cell wall using the silicon dissolved in water as orthosilicic acid Si(OH)_4_. This feature makes them one of the best studied organisms in respect to biomineralization (Kröger and Poulsen, 2008; Sumper and Brunner, 2008).

**Figure 1:**
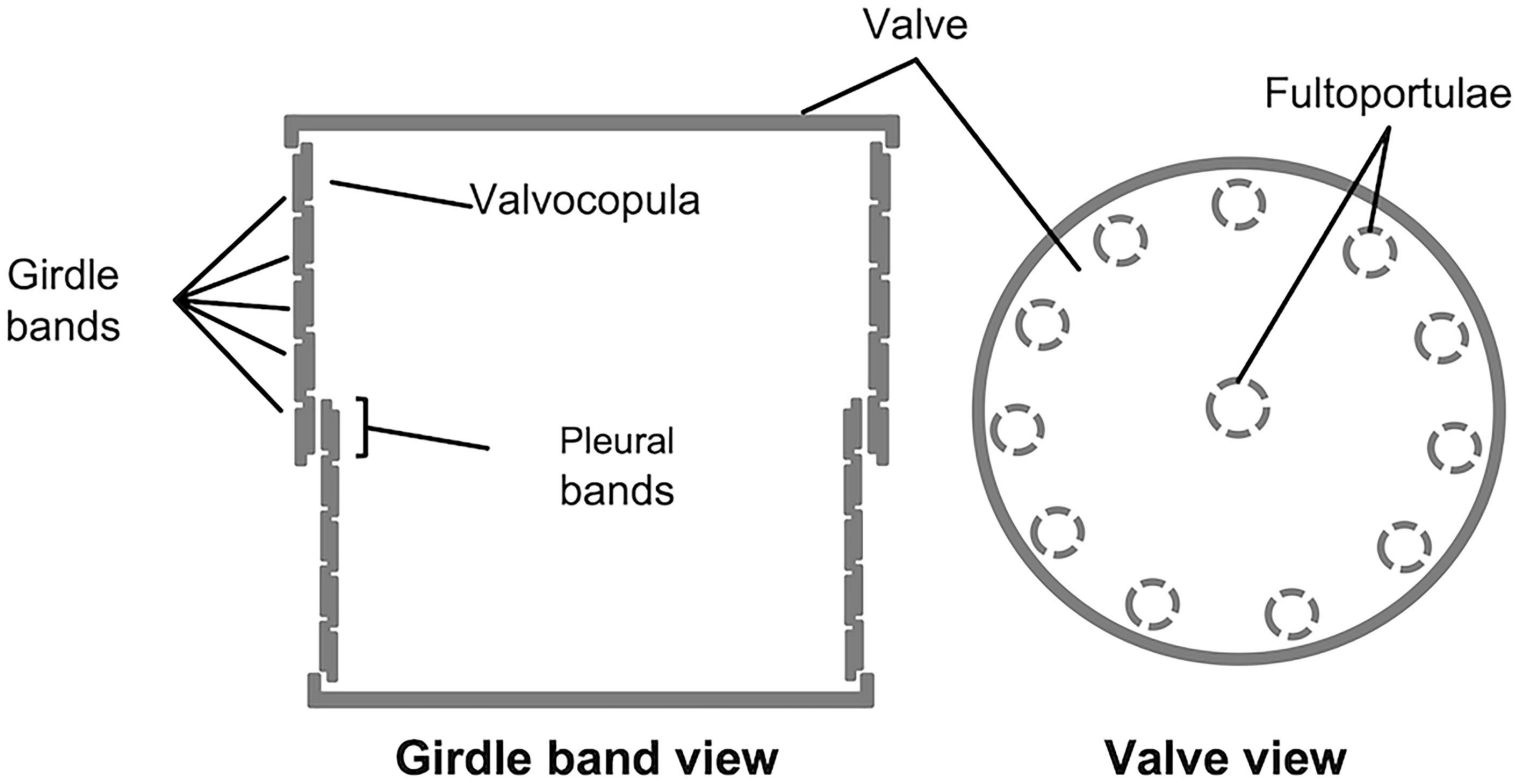
Schematic representation of *T. pseudonana*’s frustule, in girdle band view (left) and valve view (right); modified from Fattorini and Maier, 2021.

The structure of the frustule of diatoms is composed of two halves called thecae, which fit one into the other like the two parts of a shoe box (e.g., Round et al., 2007; Fattorini and Maier, 2021); the smaller one, called hypotheca, fits into the larger one, the epitheca. Each theca is made up of two main parts, the valve and the cingulum: the former, analogous to the lid (or the bottom) of the shoe box, is a somewhat flat structure, which is often extensively decorated with intricated micro- and nano-scale patterns of ribs, pores, and other silica structures; the latter, usually less decorated than the former, is connected with the valve edge and it is oriented perpendicularly in respect to the valvar plane; additionally, the cingulum is composed of a variable number of ring-like structures (the girdle bands) that are stepwise synthesized during cell growth. In each theca, the girdle band proximal to the valve is called valvocopula; whereas the distal girdle band (located in the region of the frustule where the two thecas overlap one onto the other), is called pleural band (see **Figure 1**).

The synthesis of at least some of the new components of the frustule is thought to take place in the silica deposition vesicle (SDV) (e.g., Heintze et al., 2020), a specialized cell organelle having an acidic luminal compartment surrounded by a membrane called silicalemma (Kröger and Poulsen, 2008). The formation of the new parts of the frustule is strictly interconnected with the progression through the cell cycle (e.g., Hildebrand et al., 2007; Fattorini and Maier, 2021): immediately after cytokinesis, the two daughter cells are still encased in the parental cell’s frustule; inside each cell, a valve-SDV is formed and the synthesis of the new valve starts inside it; after completion, the material for the new valve is exocytosed and becomes part of the old frustule. The daughter cells separate and grow independently, by synthesizing components for girdle bands inside girdle bands-SDV. Analogously to what previously described for the valve, when the synthesis of a new girdle band or its components is finished, the content of the SDV is exocytosed outside of the cell and becomes part of the already existing frustule (Pickett-Heaps, 1990). The formation of the SDV, the predicted biosilica synthesis steps within it, and the release of the new components via exocytosis are therefore part of a complex, highly regulated and highly refined mechanism, involving several cellular compartments and biomolecules.

*Thalassiosira pseudonana* was the first diatom species to have the genome sequenced (Armbrust et al., 2004) and since then it has become a model organism for the study of biomineralization in diatoms (Poulsen and Kröger, 2004). The frustule of *T. pseudonana* (Hildebrand et al., 2006) has a cylindrical shape, similar to a thick petri dish (**Figure 1**); the valve is therefore round and flat, decorated with silica ribs and with pores, and with several protruding silica structures called fultoportulae that are located at the periphery of the valve and often – but not always (Kotzsch et al., 2016) – also at its center; the girdle is composed of circular girdle bands, which are much less decorated than the valves but still contain pores. When *T. pseudonana*’s frustule is treated with detergent, cellular structures and membranes are separated from the frustule, which, however, remains intact (e.g., Poulsen and Kröger, 2004; reviewed in Hildebrand et al., 2018). In case the frustule fraction is next dissolved in an acidic solution of ammonium fluoride, a soluble and an insoluble fraction can be obtained: the soluble fraction consists of proteins (such as silaffins and silacidins) and long-chain polyamines (LCPA) (Poulsen and Kröger, 2004; Wenzl et al., 2008), whereas in the insoluble fraction microrings (Scheffel et al., 2011), protein- and carbohydrate-containing microplates (Kotzsch et al., 2016), and a chitin-based meshwork (Brunner et al., 2009) were identified. In the last years, many components of both fractions have been characterized, for example, in respect to their silica precipitation activity (Poulsen and Kröger, 2004; Wenzl et al., 2008; Scheffel et al., 2011; Kotzsch et al., 2016), intracellular trafficking (Kotzsch et al., 2017), or frustule location (Scheffel et al., 2011; Poulsen et al., 2013).

As long as it is known, correct intracellular targeting of proteins to the frustule is depending on specifying polypeptide sequences of the proteins to be transported (reviewed in Fattorini and Maier, 2021). However, in addition to determining the intracellular trafficking route, short signals within the proteins might also specify the frustule sub-location i.e., the valve or the girdle bands). In *T. pseudonana*, all known cell wall-associated proteins, (e.g., silaffins, cingulins, silacidins) are synthesized as pre-proteins with an N-terminal signal peptide (SP) for co-translational import into the endoplasmic reticulum (Poulsen et al., 2013); thus, after being translated, proteins initially localize in the ER lumen, or in the ER surrounding membrane in case of membrane proteins. In respect to post ER-trafficking, our knowledge is limited. However, one exception is known for silaffins, a prominent class of proteins found in the frustule. *In vivo* localization experiments showed that these proteins have different frustule locations, depending on the protein and also on what promoter has been used to drive the expression of the gene (*i.e.*, the native or an inducible, constitutively expressed one): Silaffin-1 (Sil1) located only in the valve region, specifically labelling the fultoportulae; Sil4 is found in the valve and in the proximal girdle bands (both for Sil1 and Sil4, the localization has been obtained using inducible promoters) (Poulsen et al., 2013; Poulsen et al., 2007); Sil3 is found in the valve and in the proximal girdle bands when using its native promoter (Scheffel et al., 2011), whereas it localizes in all parts of the frustule when using an inducible one (Poulsen et al., 2013). Interestingly, silaffins possess short targeting peptide regions composed of a stretch of 12-14 amino acids containing 5 lysines interspaced with other residues (named pentalysine clusters, or PLC). PLC were identified thanks to a detailed analysis on Sil3, but they were tested also in Sil1 (all silaffins known to date, in fact, possess at least one predicted PLC). These experiments, using either silaffins-derived individual PLC (PLC1 from Sil1 and PLC3ct from Sil3), or an artificially created one partially matching their consensus sequence (PLCart), showed that these motifs are involved in the targeting of eGFP-fused proteins to the frustule (although with different efficiency and specific frustule location) (Poulsen et al., 2013).

Another important group of frustule-associated proteins are the cingulins (Scheffel et al., 2011; Kotzsch et al., 2016). These proteins were shown to have a girdle location, although with slight individual differences concerning the number and position of the girdle bands labeled by eGFP-cingulins fusion proteins. As most silaffins, also the cingulins were localized *in vivo* upon expression of the genes with an inducible promoter (Scheffel et al., 2011); the only exceptions were given by the cingulins CinW2 and CinY2 that were localized also using their native promoters, and by CinY4 that was localized using its native promoter and terminator only (Kotzsch et al., 2016). The comparison between the use of inducible or native promoters for the *in vivo* localization experiments (Kotzsch et al., 2016), showed a remarkable difference in the location of CinY2: the fluorescent signal labelled all parts of the girdle when using the inducible promoter; instead, when using the native promoter, the eGFP labelled only the pleural bands. On the contrary, CinW2 did not show any different location when using the native or the inducible promoter (Kotzsch et al., 2016).

To extend our knowledge about cell-wall targeting signals in frustule-associated proteins from *T. pseudonana*, we have carried on three distinct experimental approaches: first, we have studied whether protein motifs in the cingulins CinW2 and CinY2 can be correlated with targeting activity; second, we have studied if the permutation of the promoters between the *sil3*, *cinW2*, and *cinY2* genomic sequences could affect the targeting of the corresponding encoded proteins; third, we have screened existing *T. pseudonana’s* datasets for new potential frustule-associated proteins. Here we show that at least three different signals (depending on the type of protein) are involved in specifying the localization of cingulins in the frustule, and that a cell cycle-depending expression regulation is essential for correct protein targeting in one cingulin. In addition, the screening of the available databases with the here determined targeting signals resulted in the discovery of two new frustule proteins, a result which in parallel showed the importance of the used probes for frustule localization.

## Materials and methods

### Cell cultures

The *T. pseudonana* strain CCMP 1335 (Armbrust et al., 2004) was cultivated as described in Poulsen et al., 2013. A 14/10 light/dark light regime was used for the growth of the cultures, and the value of irradiance was approximately 80-90 µE m^-2^s^-1^. To determine the cell density, cell counting was performed using a Thoma counting chamber (link 1), using approximately 8 µL of culture.

### *In silico* analysis

The genomic sequences of the predicted genes *12162* and *5357* were retrieved from the JGI database (link 2), and analyzed and managed using the software Sequencher, which was also used to generate the *in silico* DNA constructs encoding the modified Sil3-, CinW2-, and CinY2-derived polypeptides; the corresponding amino acid sequences were analyzed and managed using the Benchling online suite (link 3). The amino acid sequences of the Sil3, CinW2, and CinY2 proteins were retrieved from the corresponding NCBI deposited data (Poulsen and Kröger, 2004; Scheffel et al., 2011) and were used as templates to generate the *in silico* truncated/modified proteins. The presence/absence of signal peptides or trans-membrane domains in the predicted proteins were confirmed by the SignalP 3.0 internet tool (link 4) and by the TMHMM internet tool (link 5), respectively. For the identification of the protein Tp5357, the SLRG gene dataset was manually screened for clusters of lysines. Blast (link 6) searches of the *T. pseudonana* database using the UM led to the identification of Tp12162. Both candidate proteins were *in vivo* localized as eGFP fusion proteins.

### Cloning and sequencing

All constructs used in this work were derived from the parental plasmid pTpNAT-MCS (for the details regarding the design of this vector see **Figure S5**). As indicated in **Table S1**, the transformation vectors were created using: i) traditional cloning techniques (i.e., restriction site-based insertion); ii) site-directed mutagenesis; iii) the Gibson Assembly technique (Gibson et al., 2009). The genomic sequences of *sil3*, *cinW2*, and *cinY2* (containing both their native promoters and terminators and the *egfp* gene) were provided by Prof. Nils Kröger and cloned using the primers listed in **Table S1**. The resulting vectors pTpSil3, pTpCinW2, and pTpCinY2 have been used as templates for the creation of all their derived constructs. The genomic sequences containing the *12162* and *5357* genes, together with their promoters and terminators (approximately 800 bp and 300 bp each, respectively), were amplified from genomic DNA (DNA extraction protocol was performed as in (Manfellotto et al., 2021) and cloned into the pTpNAT-MCS plasmid. All constructs were created with the eGFP tag located at the C-ter, except for the vector pTp12162-eGFP, in which the fluorescent protein is located internally (after amino acid 410). For the promoter permutation experiments, the fragments have been exchanged using the Gibson Assembly technique (Gibson et al., 2009) (see **Table S1**). The sequencing service of the Macrogen company was used to confirm the sequences of all the DNA constructs.

### Biolistic transformation

For each transformation experiment approximately 400 million cells of an exponentially growing culture were harvested by centrifugation (12 min, at 21°C, 3200 rcf); the pellet was resuspended with 900 µL of fresh NEPCC medium and 300 µL of this solution were spread at the center of a NEPCC-based 1.5% (m/v) agar plate, for a total of three plates for each construct. For the preparation of the microparticles, 3 mg of M10 tungsten microparticles (diameter 0.7 µm) were coated with 5 µg of plasmid DNA using the CaCl_2_-spermidin method. The coated microparticles were then loaded on a Biolistic PDS-1000/He particle delivery system (BioRad) and the cells were bombarded. Immediately after, the cells were scraped from the agar plate and collected into 100 mL of fresh NEPCC medium and kept for 24 hours under constant illumination. Then, approximately 8 million cells were centrifugated and plated onto nurseothricin-containing (150 µg/mL) NEPCC-based agar plates (1.5% m/v), for a total of 10 plates per constructs. The plates were then kept under constant illumination for approximately 14 days. After that, transformant colonies were picked and transferred on nurseothricin-containing NEPCC-agar plates for microscopical observation and long-term storage.

### Confocal imaging

For the observation of live cells and simultaneous detection of the fluorescent tag and plastid autofluorescence, approximately 15 μL of cell suspension were transferred on a microscope slide and covered with a cover slip; then a drop of Leica Immersion Oil (standard and type “F”) was placed on top of the cover slip. An upright Leica DM 6000 B confocal microscope was used for the observation; the device was equipped with a HCX PL APO 63x/1.40 oil PH3 objective, a 100 mW Argon laser which could excite at 458, 476, 488, 496 and 514 nm and a DPSS that can excite at 561 nm. To excite the eGFP and trigger plastid autofluorescence, the 488 nm excitation wavelength was used, whereas two different emission detection channels were setup, one for the eGFP (500-520 nm) and one for the plastid autofluorescence (620-720 nm). All the acquired images were exported using the Leica LAS AF software, and edited using the ImageJ software (Schneider et al., 2012).

### Biosilica extraction

To isolate intact frustules of *T. pseudonana*, a slightly modified protocol was followed in respect to the previously published one (see for example Poulsen et al., 2013): roughly 4 mL of an exponentially growing culture (approximately 1.5 million cells/mL) were centrifugated at 3200 rcf for 10 min; the supernatant was discarded and the pellet was resuspended with an extraction buffer composed of 100 mM EDTA (pH= 8) and 1 mM PMSF; after the pellet was completely resuspended, SDS was added to the resuspension at the final concentration of 2% w/v; the resuspension was then kept at 55°C for 1 hour; after this step, the resuspension was centrifugated at 3200 rcf for 10 min; the supernatant was discarded and the pellet (containing extracted cell walls and degraded membranes and intracellular content) was extracted again until it was colorless. After extraction, the pellet was washed three times with 1 mL of distilled water, once with 1 mL acetone 80% and again three times with 1 mL of distilled water. The final pellet was resuspended in 20 μL and it was ready for the observation with the microscope.

## Results

### Lysine-enriched regions (LERs) in cingulins

In the proteins CinW2 (383 aa) and CinY2 (248 aa) most of the lysine residues are clustered: all 41 lysine residues in CinW2 (except for the one located in the SP) are arranged in K(X)_n_K motifs [(X)_n_ = two, three or four S or G residues], and among a total number of 20 K(X)_2_K motifs, 9 of them are clustered in regions where at least two motifs are interspaced by no more than four amino acids. In CinY2, 19 out of 22 total lysine residues are arranged in K(X)_2_K motifs, and 9 of these motifs are clustered. We have named these clusters *lysine-enriched regions* (or LERs) and identified four of them in CinW2 and two in CinY2; one of these (Y2-LER1) is a PLC, but for simplicity we will refer to it as an LER; (see also **Table 1**).

**Table 1:** Lysine-enriched regions (LERs) in the proteins studied in this work (lysine residues are in red color).

Name	Position	Sequence	Length	Protein
LER1-W2	76-87	KSGKSGSGKSGK	12	CinW2
LER2-W2	109-127	KSGKGSSSKGSKGSSKSSK	19	CinW2
LER3-W2	306-316	KSSKGSSKSSK	11	CinW2
LER4-W2	324-335	KSSKGSSSKSSK	12	CinW2
LER1-Y2	111-124	KSGKGSKSSGKSGK	14	CinY2
LER2-Y2	162-201	KSGKGSSGKSGKSSSKSSKGSGKSSKSSGKSSKSSGKSGK	40	CinY2
LER-12162	337-372	KSGKGGKGSKSGSKSAKSSSKGSKSSGKSGKSGSWK	36	Tp12162
LER-5357	148-171	KSGKGGKSSKSTKSHKSKAGKSVK	24	Tp5357

For details see text.

### *In vivo* localizations

#### Native localizations

**Figure 2:**
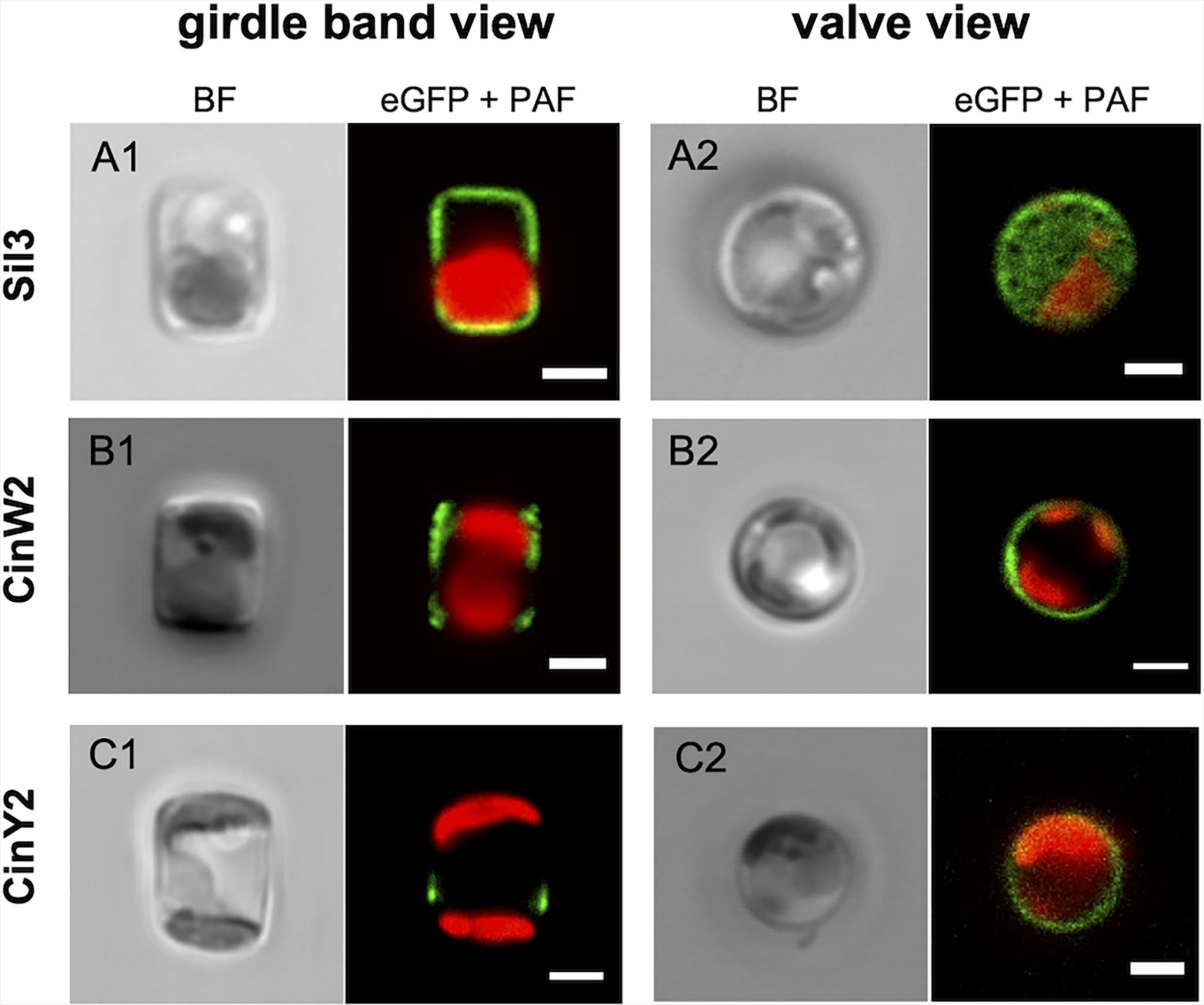
*In vivo* localization of the eGFP-fusion constructs encoding for the proteins Sil3 **(A)**, CinW2 **(B)**, and CinY2 **(C)**. The images show transapical sections of live cells in girdle band view (columns on the left) and transversal sections of live cells in valve view (columns on the right; the valve view of Sil3 is a valve surface view). For each view, the bright field channel (BF) and the merge of green and red channel are displayed, showing the eGFP and plastid autofluorescence (PAF) signals, respectively. Scale bars: 2 µm.

To avoid any secondary effect in respect to the frustule targeting of the encoded proteins, all genes or respective subclones were expressed *in vivo* using their native promoter and terminator (approximately 800 bp of the promoter region and 300 bp of the terminator); additionally, the proteins were always fused with eGFP at their C-terminus (if not otherwise specified). The results of our *in vivo* localization experiments with the eGFP-tagged native Sil3, CinW2, and CinY2 proteins (**Figure 2**) were identical to those from previously published experiments: Sil3 (**Figures 2A1-A2**) localized in the valve and in some girdle bands except for the central area of the girdle (as in Scheffel et al., 2011); CinW2 (**Figures 2B1-B2**) showed the GFP signal in several girdle bands, spanning all the girdle area (as in Kotzsch et al., 2016); CinY2 (**Figures 2C1-C2**) localized exclusively in the central part of the girdle (as in Kotzsch et al., 2016). The fluorescent imaging of the extracted biosilica confirmed these observations (see **Figure S1** in the SI).

#### CinW2-derived constructs

**Figure 3:**
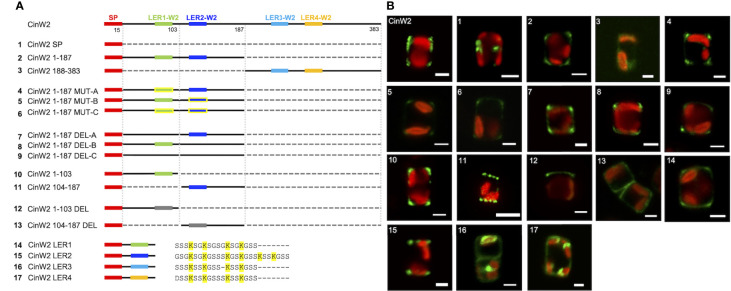
Localization experiments using eGFP-fusion constructs derived from CinW2. **(A)** shows a graphical depiction of the constructs used for the experiments. The color code is: red for the signal peptide (SP), green for LER1-W2, dark blue for LER2-W2, light blue for LER3-W2, orange for LER4-W2, black for the protein backbone, grey for the parts of the protein that have been deleted; the LERs that have been mutated have yellow contour. Next to the four constructs bearing the individual LERs is shown also the amino acid sequence following the SP. **(B)** shows girdle band view images (merged eGFP and PAF channels) of live cells selected from *in vivo* localization performed using the constructs shown in the left panel. Scale bars: 2 µm.

To study the targeting of CinW2, a series of modified constructs derived from this protein were generated (see **Figure 3A**). A first construct (CinW2 SP) bearing only the CinW2 signal peptide (SP) was localized, and the corresponding eGFP signal (image 1 of **Figure 3**) was located in some intracellular sub-compartment closely associated with the plastid (similarly to the one obtained by the SP construct derived from Sil3 used in Poulsen et al., 2013). Next, we have localized two constructs corresponding to the N-terminal (CinW2 1-187) and C-terminal (CinW2 188-383) regions of the CinW2 protein: CinW2 1-187 spans from amino acid 1 to 187 whereas CinW2 188-383 covers amino acids from 188 to 383 (in this second construct the native SP was fused at the N-terminus). As a result, the eGFP-tagged CinW2 1-187 could be detected in the frustule, with a strong signal in the area of the valvocopula and the proximal girdle bands (image 2 of **Figure 3**); this location appears similar to the native location of CinW2 (see **Figure 2B**) although in that case also some distal girdle bands are labelled. On the contrary, CinW2 188-383 was not observed in the frustule, instead a very faint signal could be detected in an intracellular location (image 3 of **Figure 3**).

As shown graphically in the left panel of **Figure 3**, CinW2 1-187 and CinW2 188-383 carry two LERs each (LER1-W2 and LER2-W2, and LER3-W2 and LER4-W2, respectively). To identify the putative targeting signal(s) that appear to be present in CinW2 1-187 and absent in CinW2 188-383, we created additional CinW2 1-187-derived constructs where the lysines of LER1-W2 and LER2-W2 were exchanged into arginines (CinW2 MUT constructs), or where the LERs were deleted (CinW2 DEL constructs). Both approaches were applied on each individual LER, and on LER1-W2 and LER2-W2 simultaneously, resulting therefore in three MUT constructs and three DEL constructs. Interestingly, none of these constructs (see images 4-9 of **Figure 3**) resulted in a mis-localization of the expressed proteins compared to that obtained by the CinW2 1-187 truncation (image 2 of **Figure 3**), from which the MUT and DEL constructs derive, suggesting no influence of these LERs on the protein targeting. The analysis of the biosilica extracts confirmed the location for constructs MUT-B, MUT-C, DEL-A, DEL-C (**Figure S1**). We have then made two additional sets of truncations of the CinW2 protein: the first set directly derived from construct CinW2 1-187 and generated the construct CinW2 1-103 (comprising amino acids from 1 to 103, therefore also including LER1-W2), and the construct CinW2 104-187 (comprising the SP plus amino acids from 104 to 187, therefore also including LER2-W2). The location of the construct CinW2 1-103 was substantially identical to those of CinW2 1-187 and its mutated or deleted derived constructs: the fluorescent labelling was located only in the valvocopula, highlighting two ring-like regions at the rims of the valves, without any significant amount of fluorescence coming from the remaining areas of the valves or the girdle (image 10 of **Figure 3**). The localization of CinW2 104-187, instead, resulted in a completely different labelling: the only area of the frustule showing fluorescent tagging was the valve-located, circular pattern resembling the organization of the fultoportulae (image 11 of **Figure 3**). The biosilica extracts of CinW2 1-103 and CinW2 104-187 confirmed the observations of the *in vivo* localizations (**Figure S1**). The second set of truncations was directly derived from CinW2 1-187 DEL-C: analogously to CinW2 1-103 and CinW2 104-187, the two constructs generated (CinW2 1-103 DEL and CinW2 104-187 DEL) covered amino acids from 1-103 and from 104-187, but they were lacking LER1-W2 and LER2-W2, respectively. Construct CinW2 1-103 DEL showed the same valvocopula-located labelling as previously observed for other constructs, albeit the signal was not only very faint and unevenly distributed in that area, but it was also apparently absent from the opposite pole of the cell; additionally, intracellular sub-compartments showed minor amounts of fluorescence (image 12 of **Figure 3**; see also **Figure S2**). Construct CinW2 104-187 DEL was instead localized mostly in the valve’s area, but a minor fraction of the fluorescent signal was also present in the girdle and inside the cell; interestingly, the fultoportulae appear to be excluded from the labelling (image 13 of **Figure 3**; see also S2). The analysis of the biosilica extracts from construct CinW2 104-187 DEL confirmed the observations of the *in vivo* localizations (**Figure S1**). Finally, to test if the individual LERs from CinW2 provide frustule targeting capacity, we have created four constructs (constructs W2 LER1-4) each one encoding for the SP of CinW2, followed by one of the four LERs of CinW2 together with the three amino acids flanking the LER at the N- and C-termini, respectively. By expressing these constructs as eGFP-fusion proteins we could show that W2-LER1 had a similar location (image 14 of **Figure 3**) respect to the one observed for CinW2 1-187 (see image 2 of **Figure 3**), whereas W2 LER-2 was localized exclusively in the fultoportulae (image 15 of **Figure 3**; see also **Figure S2**). On the other hand, both the W2 LER-3 and W2 LER-4 constructs showed intracellular location, with only a minor fraction of the signal labelling the frustule (image 16 and 17 of **Figure 3**, respectively). Additionally, as an instance, we validated the eGFP-localizations in biosilica extracts for W2-LER1, W2-LER2, and W2-LER4, resulting in the confirmation of the *in vivo* localization results (**Figure S1**).

#### CinY2-derived constructs

**Figure 4:**
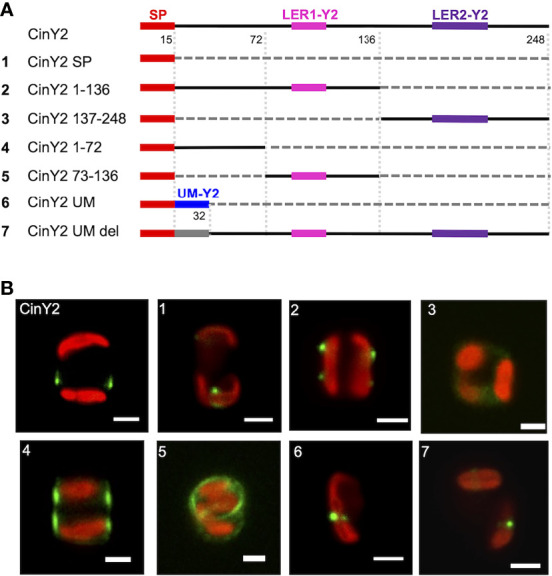
Localization experiments with the GFP-fusion constructs derived from CinY2. **(A)** shows a graphical depiction of the constructs used for the experiments. The color code is: red for the signal peptide (SP), pink for LER1-Y2, purple for LER2-Y2, black for the protein backbone, grey for the parts of the protein that have been deleted. **(B)** shows girdle band view images (merged eGFP and PAF channels) of live cells selected from *in vivo* localization experiments performed using the constructs shown in the left panel. Scale bars: 2 µm.

Similarly to what was done with CinW2, CinY2 was analyzed for targeting signals. We first localized a construct having only the SP fused to the eGFP (CinY2 SP; see panel A of **Figure 4**), resulting in a comparable location as the SP-construct of CinW2 (see image 1 of **Figure 3**). The construct CinY2 1-136 (which extends from amino acid 1 to 136 and encodes one LER, LER1-Y2) was localized in the frustule; however, the native frustule location was lost, as the signal was in two non-adjacent girdle bands, more specifically in the region of each theca comprised between the valve and the pleural bands (image 2 of **Figure 4**). As observed with the CinW2 188-383 truncation, the construct CinY2 137-248 (extending from amino acid 137 to 248 and encoding LER2-Y2 plus the SP) could not be observed in the frustule, instead a faint fluorescent signal appeared to be retained in an unspecific intracellular location (Image 3 of **Figure 4**). To test which region of the construct CinY2 1-136 provides targeting activity, an additional set of subclones was created, generating the constructs CinY2 1-72 and CinY2 73-136 (the latter encoding CinY2-LER1). The *in vivo* localization of CinY2 1-72 and CinY2 73-136 showed that both have frustule targeting capacity, although the specific location in the frustule is different, not only between the two constructs but also between these two constructs and the native location of the CinY2 protein (see **Figure 2C**): the construct CinY2 1-72 gave a dual, symmetrical localization that appears to tag the central part of each theca (image 4 of **Figure 4**); also the construct CinY2 73-136 resulted in a dual localization in which the GFP signal labelled the rim of the valve or the first girdle band (image 5 of **Figure 4**); interestingly, a faint signal could be observed in the fultoportulae of one valve only, whereas on the other valve there was a girdle band-like signal. The observation of the biosilica extracts from CinY2 1-72 and CinY2 73-136 confirmed the frustule localization observed in the live cells (**Figure S1**). In any case, the data generated with construct CinY2 1-72 demonstrated that this CinY2-derived fragment is targeted to the frustule via some LER-independent mechanism. Thus, another targeting signal should be present in the portion of the N-terminal, LER-free, region of CinY2 following the SP. In fact, we have identified a 17 amino acids-long peptide-sequence of CinY2 which is located immediately after the SP and is conserved among the three Y-type cingulins CinY1, CinY2, and CinY3 (both for its position and its sequence; see **Figure S4** and amino acid sequences in the SI). This conserved region was named *unknown motif* (or UM; see **Figure S4** and amino acid sequences in the SI) and was tested for a possible influence in ensuring the correct targeting of the protein CinY2. To do so, we have constructed two new constructs deriving from the full-length CinY2 (see constructs 6 and 7 in panel A of **Figure 4**): the first construct (CinY2 UM) expressed the CinY2 SP followed by the UM only, and its *in vivo* localization showed that the eGFP signal was entirely retained inside the cell, with no region of the frustule tagged (image 6 of **Figure 4**); the second construct (CinY2 UM DEL) was instead created by deleting only the UM from CinY2, and the localization experiment using this construct (image 7 of **Figure 4**) showed a similar result to that obtained by the localization of the CinY2 UM, suggesting a targeting function of the UM.

#### New frustule-associated proteins

**Figure 5:**
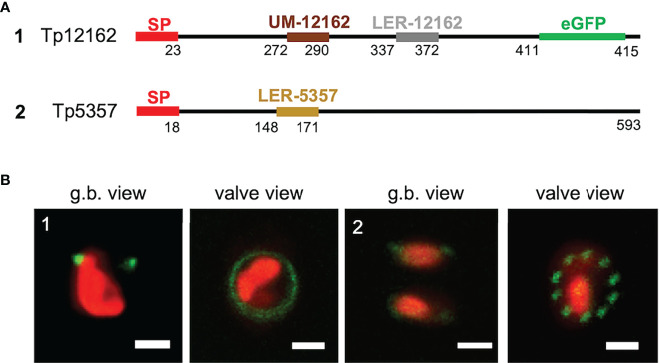
Localization experiments with the Tp12162 and Tp5357 eGFP-fusion proteins. **(A)** shows a graphical depiction of the constructs used for the experiments. The color code is: red for the signal peptide (SP), brown for the UM-12162, grey for LER-12162, ochre for LER-5357, black for the protein backbone; the eGFP is shown only for construct Tp12162-eGFP, as its position is internal. **(B)** shows images (merged eGFP and PAF channels) of live cells selected from *in vivo* localization experiments performed using the constructs shown in the upper panel. Scale bars: 2 µm.

LERs and the UM motif appear to have influence on the frustule targeting. Thus, they might be present in other, still not characterized proteins. We have screened the *silaffin-like response genes* (SLRG) dataset (Shrestha et al., 2012; link 7), containing 485 genes whose expression was regulated similarly to the *sil3* gene, as well as the *Thalassiosira pseudonana* database (link 2) for potential, yet unknown frustule-located proteins. For this screening, the following criteria were applied: i) presence of a SP; ii) absence of any trans-membrane domain; iii) presence of LER-like region(s) or of an UM motif. Two proteins, matching the criteria, were investigated in more detail: Tp5357 and Tp12162. Tp12162 was identified by the screening with the UM; according to the *in silico* predictions, this protein is 415 amino acids long and has an UM motif located approximately in the central part of the amino acid sequence (after amino acid 271) and not immediately after the SP as it is in the three Y-type cingulins CinY1-3 (see also **Figure S4**); Tp12162 carries also a 36 amino acids-long LER (see **Table 1**) and a so-called RXL motif (after amino acid 410) which is known as a potential target site for proteolytic cleavage (see for example Poulsen et al., 2013). To avoid undesired cleavage, eGFP was fused immediately upstream of this RXL motif (construct 1 of **Figure 5**). The *in vivo* localization of the Tp12162-eGFP fusion protein showed fluorescent labelling of the frustule, with the fluorescent signal tagging a ring-like structure in the girdle (most likely one or few girdle bands; image 1 of **Figure 5**). The observation of the biosilica extracts confirmed that the Tp12162-eGFP signal was in the frustule (see **Figure S1**). The protein Tp5357 was selected upon the screening of the *SLRG* dataset (Shrestha et al., 2012); the predicted protein is 593 amino acid long, has a 24 aa-long LER after amino acid 147 and also two RXL motifs. In this case, the eGFP tag was fused directly to C-terminus of Tp5357 (construct 2 of **Figure 5**). The *in vivo* localizations of the Tp5357-eGFP fusion protein showed frustule labelling in the valve; more specifically, the fluorescence tagging pattern strongly resembled the position pattern of the fultoportulae (image 2 of **Figure 5**).

#### Promoter studies

**Figure 6:**
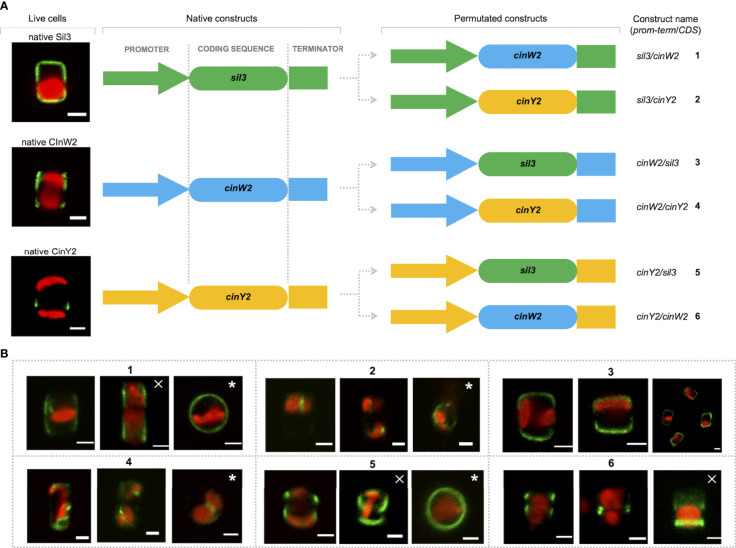
Localization experiments with the constructs obtained upon permutation of the regulatory regions between the *sil3*, *cinW2 and cinY2* genes. **(A)** shows a graphical depiction of the constructs used for the experiments, highlighting the substitution of the native coding sequences of each gene with the coding sequences of the other two genes, therefore resulting in six new constructs (the position of the *egfp* coding sequence is omitted for simplicity, as it is the same as in the parental Sil3-eGFP, CinW2-eGFP or CinY2-eGFP constructs). **(B)** shows girdle band view images (merged eGFP and PAF channels) of live cells selected from *in vivo* localization experiments performed using the constructs shown in **(A)**. The images marked with a cross are Z-stack projections; the images marked with an asterisk are in valve view. Scale bars: 2 µm.

For all localization experiments described above, the respective native promoters/terminators were used. According to Hildebrand et al., 2018, the *cinY2* mRNA expression maximum is in G2/M-phase whereas *cinW2* expression is upregulated in the pre-valve/valve phase (as defined by Hildebrand et al., 2018). Notably, the expression maximum of *cinW2* is similar to one of the two maxima of *sil3*, the model silaffin. To test if the use of non-native promoters can affect the targeting of the encoded proteins, we performed promoter swapping between the *sil3*, *cinW2*, and *cinY2* genomic regions, and localized *in vivo* the corresponding proteins. To do so, six additional expression constructs were created, in which the coding sequence (CDS) of each one of the three genes was flanked by the promoter/terminator regions of the other two genes (see **Figure 6A**): the two constructs expressing the *sil3* CDS using the *cinW2* or *cinY2* promoter/terminator were named *cinW2*/*sil3* and *cinY2*/*sil3*, respectively; analogously, *sil3*/*cinW2* and *cinY2*/*cinW2* are those constructs expressing the *cinW2* CDS with the *sil3* and *cinY2* promoter/terminator, respectively, and *sil3*/*cinY2* and *cinW2*/*cinY2* are those expressing the *cinY2* CDS with the *sil3* and *cinW2* promoter/terminator, respectively. The *in vivo* expression of the above-described constructs resulted in different protein localizations, not only depending on the CDS expressed but also on the promoter/terminator used to drive its expression. In case of *sil3*, when the gene is expressed by the *cinW2* promoter/terminator (construct *cinW2*/*sil3)*, the resulting eGFP fusion protein showed an identical location (images in box 3 of **Figure 6**) as in the experiments using the native promoter/terminator of *sil3* (see image A of **Figure 2**). When using the *cinY2* regulatory regions (construct *cinY2*/*sil3*), the Sil3 protein was no longer localized in the valve (images in box 5 of **Figure 6**); the fluorescent signal tagged instead two distinct areas of the girdle, resembling two distinct non-adjacent girdle bands (or perhaps two non-adjacent groups of girdle bands) one of which was close to the valve. When the *cinW2* gene was expressed using the *sil3* regulatory regions (construct *sil3*/*cinW2*), the encoded protein localized in the girdle area only (images in box 1 of **Figure 6**) and, despite a minor fraction of the fluorescent signal was retained inside the cell, the overall frustule-targeting appeared to be identical to the one of the native CinW2 (see **Figure 2B**). When using the *cinY2* promoter/terminator regions (construct *cinY2/cinW2*), the CinW2 protein localized only in what appeared to be two distinct girdle bands (or two distinct groups of girdle bands; images in box 6 of **Figure 6**), similarly to what observed for the *cinY2/sil3* construct, although in this case one of the two regions labelled showed a less pronounced and more diffuse fluorescence respect to the other one. Regarding the set of permutations using the *cinY2* CDS, both when the *cinY2* gene was expressed using the *sil3* promoter/terminator cassette (construct *sil3/cinY2*; images in box 2 of **Figure 6**) or the *cinW2* promoter/terminator cassette (construct *cinW2/cinY2*; images in box 4 of **Figure 6**), the screening of the resulting transformants was particularly difficult, as transformation efficiency was very low and the few eGFP-positive clones exhibited faint fluorescent signals. In any case, for both constructs, the fluorescent signal resulted mostly confined in some unknown intracellular compartment and, only for construct *cinW2/cinY2*, also in the cytosol.

## Discussion

As mentioned, for most of the targeting experiments shown in this study, the proteins of interest are fused with the eGFP at their C-terminus; therefore, the signal peptide located at their N-terminus of the proteins is not masked; according to our experience, C-terminal eGFP does not interfere with the *in vivo*-localization of the examined proteins, as long as C-terminal targeting signals are not present. Our results indicated several signals involved in the correct cell wall targeting of at least Sil3 and the cingulins CinW2 and CinY2 from *T. pseudonana*. However, not only targeting signals are involved, but also a defined timing of expression was shown to be necessary for the correct localization of CinY2. This was already indicated by comparison of the localization patterns obtained by using the native and a constitutively expressed promoter in case of CinY2 and CinW2 (Kotzsch et al., 2016). With our studies, we have extended the knowledge about the correlation between the expression of genes and the *in vivo* localization of the encoded proteins by promoter permutations using *sil3* (having two mRNA expression peaks; Frigeri et al., 2006; Hildebrand et al., 2018), *cinW2* (having a minor peak overlapping with one of the *sil3*-peaks; Hildebrand et al., 2018) and *cinY2* (cell cycle phase specific expression of mRNA; Hildebrand et al., 2018). With these studies we provided evidence that the *sil3* promoter/terminator used for the expression of the *cinW2* gene, as well as the expression of the *sil3* gene via the regulatory regions of *cinW2* (constructs 1 and 3, respectively ), did not significantly change the location of the respective encoded proteins in comparison to the expression by their native regulatory regions (see **Figures 2A, B**). On the other hand, when the *cinY2* gene is expressed during the expression time frame of *sil3* or *cinW2* (images in box 2 and box 4 of **Figure 6**, respectively), we rarely obtained clones showing the eGFP fluorescent signal, and in those few cases the localization was intracellular. In agreement with the differences indicate above, the expression of *sil3* and *cinW2* by the *cinY2* promoter/terminator narrowed down the localizations of the Sil3 and CinW2 proteins to some girdle bands. Thus, the need of cinY2 for a specific gene expression time frame to ensure the correct protein destination could be interpreted as indirect evidence of an analogously specific intracellular transport step for the protein CinY2; it's possible that this specific pathway is not shared with the proteins Sil3 and CinW2, as both of them are found in the frustule although their genes are expressed at the "wrong" time. However, we cannot exclude that clearance by specific degradation of proteins which are expressed with the wrong timing plays a major role in ensuring the correct localizations of the transported proteins. For that, a possible underlying mechanism could be the re-translocation of the newly synthesized protein from the ER lumen into the cytoplasm, then followed by degradation of the protein after poly-ubiquitination. Equally likely, specifying factors with exact temporal expression might influence the protein’s localization. In any case, our experiments demonstrated that the presence of CinY2 in the frustule, and its specific localization, are strongly dependent on the cell cycle phase in which the *cinY2* gene is expressed. All known cingulins investigated to date are localized in the girdle (Scheffel et al., 2011; Kotzsch et al., 2016). To investigate the signal-dependent targeting of these cell wall proteins we have first screened their amino acid sequences for putative frustule-targeting signals, like the PLC identified in silaffins (Poulsen et al., 2013); cingulins, however, encode for LERs in which lysines are the dominant amino acids as it is in the PLC from silaffins, although their length and structure are not identical to those of the PLC. The experimental design that we have followed to study the frustule targeting of the cingulins CinW2 and CinY2 was conceived to test the *in vivo* localization of eGFP fused to sub-fragments of the protein sequences. In case of the construct CinW2 1-187 (image 2 of **Figure 3**), the observed location of the eGFP-fusion protein was obtained which was very similar to that of the wild type protein (**Figure 2B**); therefore, essential targeting was provided by the N-terminus of CinW2. Contrary to this, the construct CinW2 188-383 showed intracellular localizations together with a eGFP signal surrounding the cell (image 3 of **Figure 3**). The latter might be caused by secretion, in which the signal peptide directs the protein into the ER, thereby entering the “default” pathway of secretion as no additional targeting signals specify further intracellular pathways. The CinW2 protein carries four LERs and, as a result of the truncation, in the CinW2 1-187 only two LERs (LER1-W2 and LER2-W2) are present (see **Figure 3A**). It appears that the presence of LERs in the N-terminus of CinW2 is substantially irrelevant in respect to the frustule targeting, as the truncated protein was still localized in the frustule although the LERs of this polypeptide were either mutated or deleted (see constructs MUT and DEL in **Figure 3** and corresponding localizations (images 4-9 of **Figure 3**). This observation excludes the possibility that the different targeting properties of the CinW2 1-187 and CinW2 188-383 fragments were solely due to the targeting activity of LER1-W2 and/or LER2-W2. However, although deletion or mutation of these two LERs had no direct effect on the frustule targeting, the fact that both LERs, when expressed individually, showed frustule targeting activity (see constructs W2 LER1 and W2 LER2; and corresponding localizations in the images 14 and 15 of **Figure 3**), indicated a sophisticated targeting pathway. All the more so as individual LER constructs targeted the eGFP to the valvocopula (W2 LER1) or in the fultoportulae (W2 LER2). In any case, the targeting activity might also be influenced by non-LER sequence regions, and if an intermediate valve location in the targeting of the girdle band-specific CinW2 protein is necessary or not (as suggested by the localization of the W2 LER2 construct) still has to be verified. Similarly to the experiments with CinW2, the N-terminal region of CinY2 (construct CinY2 1-136) was expressed separately from the C-terminal part (construct 137-248), each fragment harboring only one of the two predicted LERs of CinY2. As in CinW2, only the N-terminal region of CinY2 is targeted to the cell wall when expressed as a GFP fusion protein (image 2 of **Figure 4**). However, the localization pattern of CinY2 N-terminus is slightly different to that of the full-length protein (see **Figure 2C**), which indicates that at least one further targeting signal is used for correct localization of CinY2. Thus, these results speak for a multicomponent, perhaps hierarchical system of targeting signals, in which at least one signal determines the targeting route in general and – again at least – another signal might be responsible for fine targeting. The localization experiments of two additional sub-fragments of the CinY2 1-136 construct (CinY2 1-72, CinY2 73-136) suggested the presence of several targeting signals, as both fragments could still achieve to target the eGFP to the biosilica (image 4 and 5 of **Figure 4**, respectively), although with significant differences compared to the native localization of CinY2 (**Figure 2C**). In any case, as no LER is encoded in CinY2 1-72, we searched for another signal present in this region of the CinY2 protein. The expectation of a new signal was encouraged by the alignment of the amino acid sequences of CinY1, CinY2, and CinY3 that revealed in the three proteins the presence of the UM, a highly conserved amino acid stretch immediately following the predicted SP. Again, the complexity of the cellular targeting model for CinY2 was indicated by the lack of any observable frustule targeting activity when expressing the sole UM (construct CinY2 UM; image 6 of **Figure 4**), but also when expressing the full-length CinY2 without the UM (construct CinY2 UM DEL; image 7 of **Figure 4**), highlighting its importance for the correct frustule targeting. However, we cannot exclude that further sequences immediately downstream of the UM are additional parts of this signal, which might explain the lack of frustule targeting of the here defined UM. In any case, we have identified in cingulins two new targeting signals, LERs and the UM. To test if both are exclusively present in the known cingulins, or in other frustule proteins as well, we screened two different databases and indeed identified two proteins having regions of their coding sequences resembling the LERs or the UM. These proteins were expressed as eGFP-fusion proteins and the results of their *in vivo* localization experiments showed that both are targeted to the frustule (**Figure 5**). Therefore, both signals can be correlated with frustule proteins.

## Conclusion

In respect to the cellular targeting of frustule proteins in diatoms, several models were proposed. Cingulins, frustule proteins with girdle band specificity, were here investigated as model proteins to study the mechanism underlying their targeting to the frustule. Although three targeting signals (SP, LERs, UMs) – as well as the influence of the temporally controlled expression on the targeting of cingulins – were identified, the spatial sequence of events together with further targeting motifs has to be investigated in more detail. This will not only allow to better understand the complex targeting of these frustule proteins, but also to manipulate the targeting mechanisms and, in parallel, to re-design the phenotype of a diatom frustule.

## Data availability statement

Publicly available datasets were analyzed in this study. This data can be found from: (Link 2) https://mycocosm.jgi.doe.gov/Thaps3/Thaps3.home.html and (Link 7) http://www.ncbi.nlm.nih.gov/%20geo/query/acc.cgi?acc=%20GSE37081. The additional internet references cited in the article can be found here: (Link 1) http://insilico.ehu.eus/counting_chamber/thoma.php, (Link 3) https://benchling.com, (Link 4), https://services.healthtech.dtu.dk/service.php?SignalP-3.0, (Link 5), https://services.healthtech.dtu.dk/service.php?TMHMM-2.0, (Link 6) https://blast.ncbi.nlm.nih.gov/Blast.cgi.

## Author contributions

UM conceived the project. NF and UM designed the experiments, analyzed the data, and wrote the paper. Experiments were done by NF. All authors contributed to the article and approved the submitted version.

## Funding

This work was supported by the Deutsche Forschungsgemeinschaft (DFG) through Research Unit 2038 “NANOMEE” (Ma 1232/17).

## Acknowledgments

We acknowledge steady support from Dr. Stefan Zauner, Marburg. Additionally, we thank Nicole Poulsen and Nils Kröger (Dresden) for materials and technical support.

## Conflict of interest

The authors declare that the research was conducted in the absence of any commercial or financial relationships that could be construed as a potential conflict of interest.

## Publisher’s note

All claims expressed in this article are solely those of the authors and do not necessarily represent those of their affiliated organizations, or those of the publisher, the editors and the reviewers. Any product that may be evaluated in this article, or claim that may be made by its manufacturer, is not guaranteed or endorsed by the publisher.
