# Supplementary material for: Targeting motifs in frustule-associated proteins from the centric diatom Thalassiosira pseudonana
Source: Front Plant Sci. 2022 Oct 28;13:1006072. doi: 10.3389/fpls.2022.1006072 (PMC9650480; doi:10.3389/fpls.2022.1006072)
Supplement: Supplementary file 1 [file DataSheet_1.pdf]

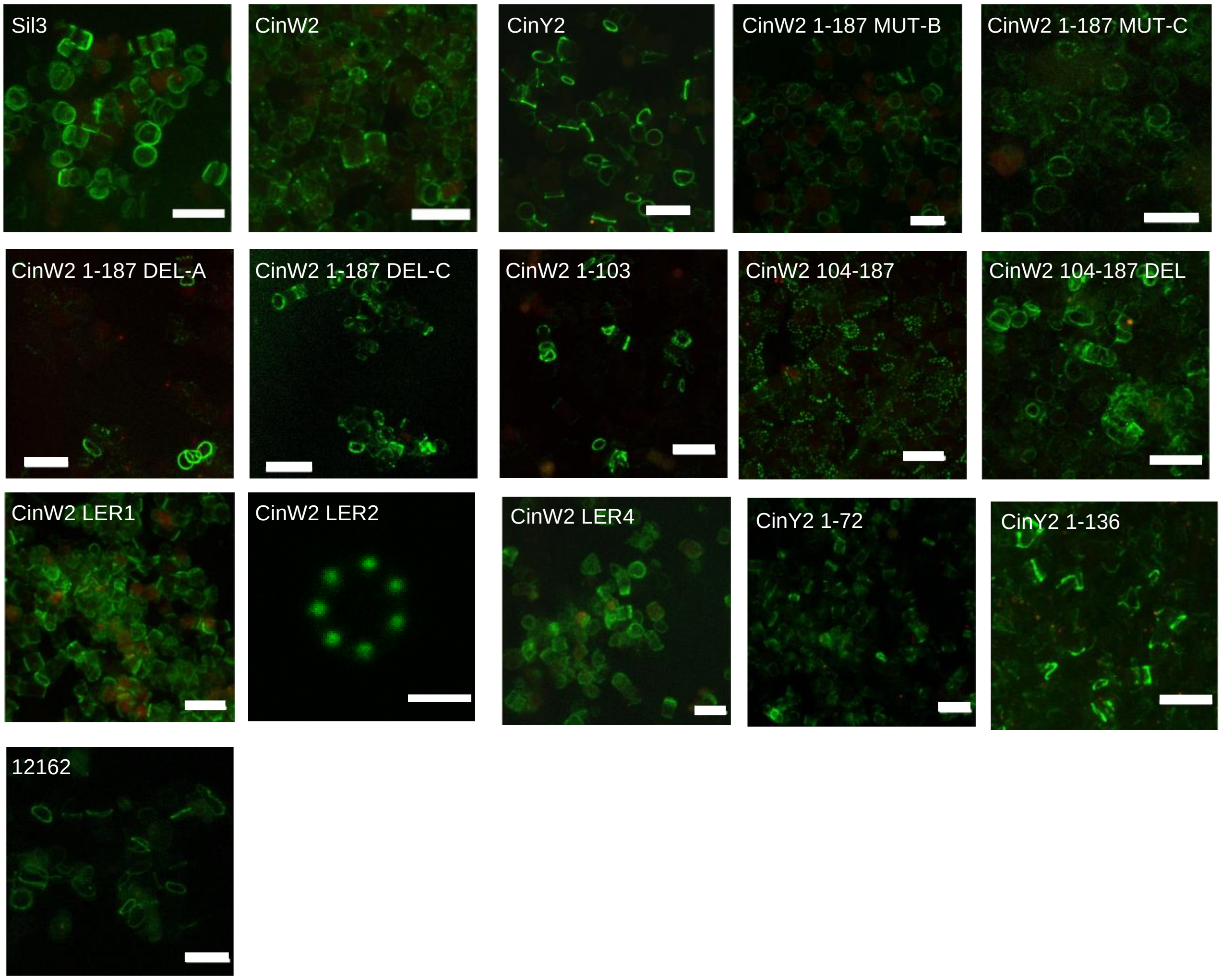

Figure S1 Confocal images of the biosilica extracted from live transformants. The images have been created by merging the green (eGFP) and red (PAF) channels. Scale bars are 10 µm (except for CinW2 LER2 where it is 2 µm).

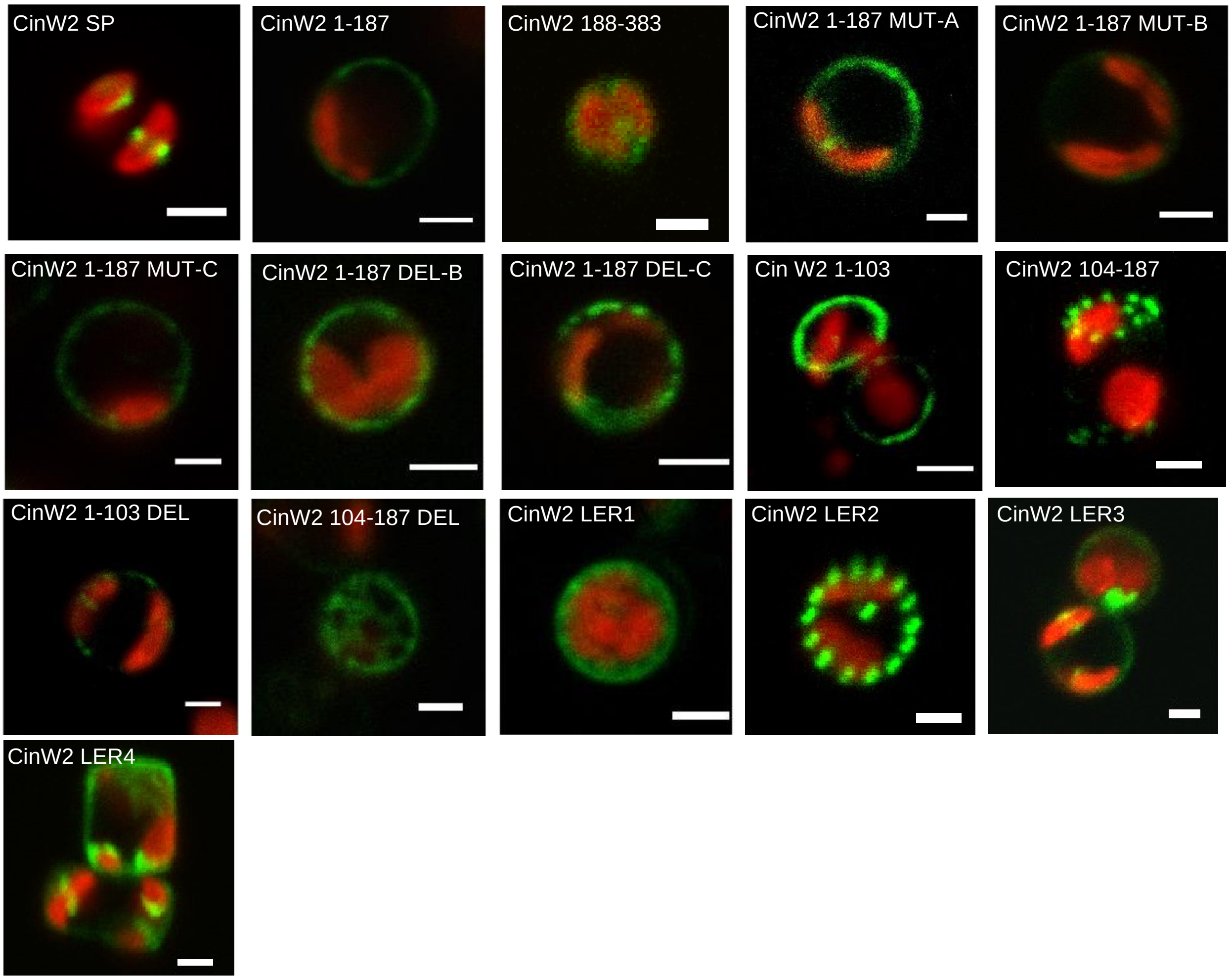

Figure S2 Additional confocal images of *in vivo* localization experiments of the CinW2-derived constructs. Each image is a merge of the red (PAF) and green (eGFP) channels. CinW2 1-103 and CinW2 104-187 are Z-stack projections of tilted cells. Scale bars are 2 µm

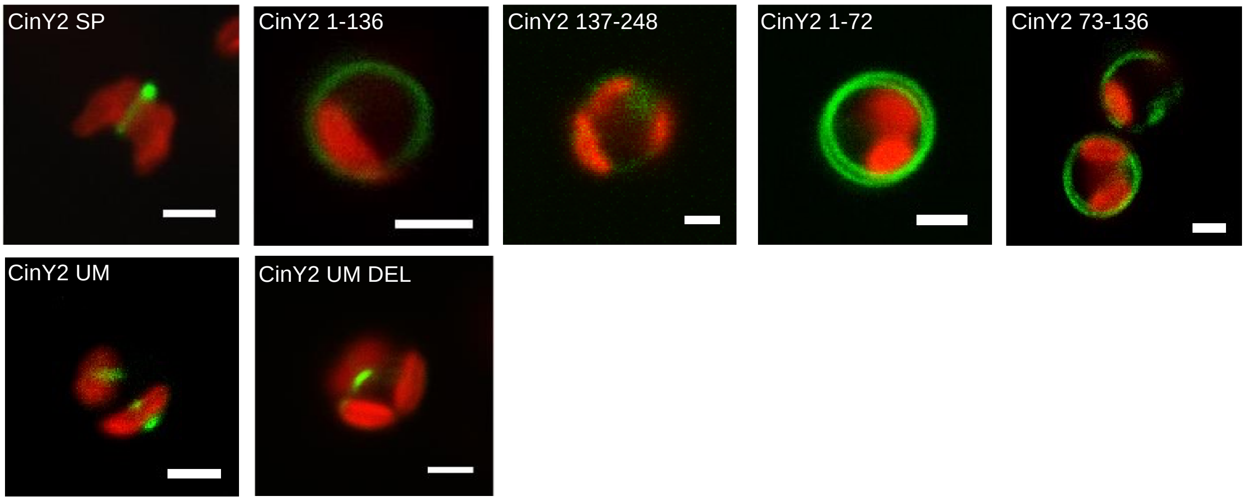

Figure S3 Additional confocal images (in valve view) of *in vivo* localization experiments of the CinY2-derived constructs. Each image is a merge of the red (PAF) and green (eGFP) channels. CinY2 1-72 is a Z-stack projection of a tilted cell. Scale bars are 2 µm

```
>UM_Y2   GTNKTLPPTPFPGRPTP 17
>UM_Y1   GTNKTLAPTPFPGRPTP 17
>UM_Y3   GTNKTLAPTPFPGRPTP 17
         ****** **********
```

```
>UM_Y2      GTNKTLPPTPFPGRPTP 17
>UM_12162   GTNKTLTPTVTPLRPTP 17
            ****** **  * ****
```

Figure S4 Alignments of the *unknown motif* (UM) in the cingulins CinY1, CinY2, and CinY3 (left) and in CinY2 and in the predicted protein 12162 (right).

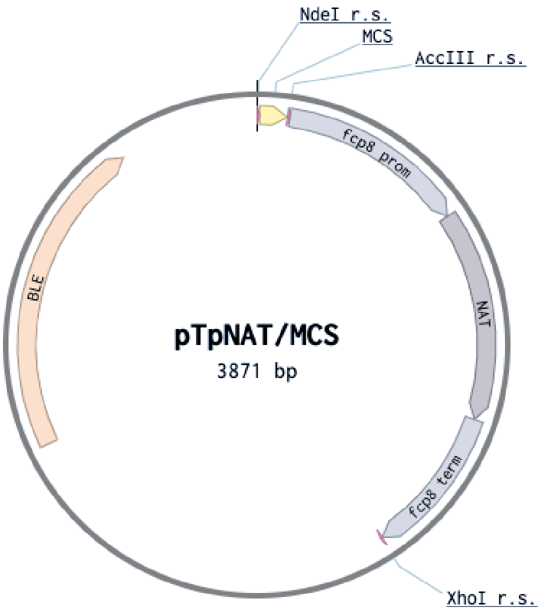

Feature	Position	Length
MCS	7-81	75 bp
fcp8 promoter	88-600	513 bp
nat CDS	601-1169	569 bp
fcp8 terminator	1170-1578	409 bp
ble	2629-3488	860 bp

Figure S5 Structure of the parental vector pTpNAT-MCS. This plasmid was designed to have: i) a multiple cloning site (MCS) for the insertion of fragments of interest; ii) the NAT (*nurseothricin acetyl transferase* gene) resistance cassette to ensure resistance to the antibiotic nurseothricin. This cassette is made of the *nat* gene flanked by the promoter and terminator of the light-induced *fcp* gene (*fucoxanthin chlorophyll a/c binding protein*; Poulsen et al., 2006).

The plasmid pTpNAT-MCS was created as follows: the first step was to amplify the MCS from the vector pPhaNR (Stork et al., 2012) using the primers pPhaT1MCSfw and pPhaT1MCSrv (that inserted an NdeI r.s. and an AccIII r.s., respectively; see Tables S1 and S2). The vector pPhaNR and the amplified MCS were then digested using the restriction enzymes NdeI and AccIII, creating two fragments bearing compatible sticky-ends; these two fragments were ligated together creating an intermediate vector having the desired MCS, but also the undesired zeocyn resistance cassette from pPhaNR. The following step was the amplification of the NAT cassette from vector 937 (Kotzsch et al., 2016) using the primers Fcp9promFw and Fcp9termRv2 (that inserted an AccIII r.s. and XhoI r.s., respectively; with these primers, the extension of the promoter and terminator regions were reduced to 500 bp and 400 bp, respectively). The intermediate vector and the amplified NAT cassette were then digested with the restriction enzymes AccIII and XhoI, generating two fragments bearing compatible sticky-ends. The ligation of the two fragments generated the vector pTpNAT-MCS. This vector was then used as parental plasmid for the creation of all the other constructs.

Amino acid sequences

For each protein is indicated the name (if available) and/or the corresponding JGI protein ID, together with the total number of amino acids. For each protein, the predicted signal peptide is in red bold, all lysine residues (except for those in the SP) are highlighted in yellow, the LERs or the PLC are underlined, other specific features are highlighted in color.

CinW2 (ID: 25075) 383 aa

MKLALFLTIPTLIAAQQSSVRGVATTSSRQLDEWGDDAWGSSDSGSSGKSGKSGGSASSGDGWETD
GWGGDYSSSKSGKSGSGKSGKGSSGPHGHWVYIEDDSSDGSGKSGKGSSSKGSKGSSKSSKGSSSD
DSTDDSWDGGWGGHGGWNGDNSGKSGKGSYGSGKSGKGSSYPSSHWGPSHWGSDDDDSSSSKSSKG
SSESSSKSSKGSSDSSSKSSKGSSSSEDEGHWEWEGGYGSGKSGKGSYSGSSGKSGKSGSGDSWVG
DYGSSGKSGKGSYGGDSWGGNYNGWGGHYDVDVDDDDSSSSKSSKGSSKSSKGSSEDSSKSSKGSS
SKSSKGSSSSEDEGHWVWEGSYGSGKSGKGSYSGSSGKSGKSGSGDEGWYSGW

KSGKSGSGKSGK LER1-W2
KGSKGSS-KSSK LER2-W2
KSSKGSS-KSSK LER3-W2
KSSKGSSSKSSK LER4-W2

CinY2 (ID: 25910) 248 aa

MKLIIALSAITLASAGTNKTLPPTPFPGRPTPNPTMVNTIGTPGPSFIVTEQTPAPTPGDVLTPQP
TPLPTLGGVPTTKMPTEMSYGYGYGDYGIVDCFGKSGKSGSGCGKSGKGSKSSGKSGKSGGGGGG
YGYGDNYADDYTPSTDDYEYGYGHGGSSGKSGKGSSGKSGKSSSKSSKGSGKSSKSSGKSSKSSGK
SGKGGSRDDGHGYGGYGGYEGYGGYEGYQYGGDEYVRRNRRLGASHNNRI

KSGKGS--KSSGKSG-K LER1-Y2
KSGKGSSGKSG-KSSSKSSKGSGKSSKSSGKSSKSSGKSGK LER2-Y2

GTNKTLPPTPFPGRPTP UM-Y2

CinY1 (ID: 12168) 280 aa

MKSIIALSTIALASAGTNKTLAPTPFPGRPTPIPTPVNTYIVTEQTPAPTPGDVITPAPTICEEKI
FFFDGGMCTNMFEVADGSSYNTLIQCCNANFGSFAMCVYEDMCVDVKPTRRPTTRPPTDMSYNYGI
VDCFGKSGKSGSGCGKSGKGSKSSKSGGGYGYGDNYVDDYTPSTNDYSHSTNDYTPSTNDYEYGYG
HGSSGKSGKGSKSSKGGKSSKSSGKSSKSSGKSGKGSSSSGKSGKGSDGHYTGDGYRYDDDAYYRK
LSEGQAGGLRRTRKMP

GTNKTLAPTPFPGRPTP UM-Y1(identical to UM-Y3)

CinY3 (ID: 25917) 352 aa

MKFSASILLLTVATASAGTNKTLAPTPFPGRPTPPGAGTPFPTENTPAPSPAFGTKPPTPVSESVA
SLLIVSWFVLGSMWPLNGRMNVSLTVHTVDRWRADTTLKDGTAEQRDRCSSYEPPQYSYEPPTTGC
SKAGKGGKSGSMDYLIDCIDLSSKSGKSGSGYGPSSSKGGKSGSSSAGYGDDYTATTDDYSAGADA
GKSENYDEEASRDDGHYGASSKGGKSGSAGYGDEGYGSSAGSSKGGKSEADGYGDESYGDSGDSKA
GKAEAGYGDDYGASAKSGKGSDGYGSSSKSGKAGSAKSGKGEGYHMFHDKSGKGGKGSSSGGEGYG
YGYDEAHDYGYGRRTRGLRASQ

GTNKTLAPTPFPGRPTP UM-Y3(identical to UM-Y1)

Tp5357 (593 aa)

MRVLCLLAFLQVTSFAVSNEVDSELPSRKHLLRNTRDPQQRSLQQKEYRLVKPKDRRVVRKERNSK
LAREDDGERTEVIPELERASNGGTFEFEVDDEESVNVVVTVNVETTATEEEEPSEDVDEMCPEWEI
VYVKQIQHQHHHASKSGKGGKSSKSTKSHKSKAGKSVKEVPVKKCKTTKPTSKPAPVTPMPIGGA
TPIPTMANITPDPTDVPPTSTTSSPTKAGDNITSNPTIDSIETSAPVASAPPNPVPTDVPVLAPTM
PPMSGPLPTLPPAEATSSPTVSVPQPTDVPISVAPTMPPMSGPLPTLPPAEATSSPTVSVPQPTDV
PISVAPTMPPMSALTSAPVATGSVSPTVNNQTSASPTVNNETIVTDAPSAAPSTTTATDAPSVSAA
PSTGEKQPTESPTYMPSYIPTKAPVAGIVPSTAPTTGSGLTTLSWDFEDQQFPIDPWTTGGDGVWA
IDDTNADGGSVYSLKSPDLEDDTNPSPQLSNATLTLSDTFAGGVMRMRVLASVLPPRDIFVVYIDG
ESAAQLVDVQEFTDVVLGVGPGAHVIDFSYQYNFFGVDPLPPSPPSRIGSVWIDNVSIETLAAVP

KSGKGGKSSKSTKSHKSKAGKSVK 5357-LER

Tp12162 (415 aa)

MKHHAILLTALLALSLTTPSVEAADSNPTKKNIKAVKKLLRKKIRKQRKKNMKEDAATNAAATTTT
STAAAVVSKNNTIDGNKPTWSPTMWPTYWPTYSPTNEKMSEETGESIPVSAPVTKQPTPKPSKAPT
PSPSKAALVQSQVEIPVANPDSAQTNLNNLVFISVLVNDVPAMGQSLDVFAIASNAANGNCAVSLN
IQQVVYTPNDGFVGFDECTYEACDGMEMCDTAIVTIEVLKEENTPVPTFANTRVNPTPFPSQDVIT
FEPTIASGTNKTLTPTVTPLRPTPFPTENTPAPTFIVDTPTTRLPTGYTDEKYYTDDYGYSMPYLS
HIDLSSKSGKGGKGSKSGSKSAKSSSKGSKSSGKSGKSGSWKGDMHGDYGDDDGYTFDDGYAHDDG
WYGGEAGGKSGKGRRMLRQ

KSGKGGKGSKSGSKSAKSSSKGSKSSGKSGKSGSWK 12162-LER
GTNKTLTPTVTPLRPTPFP UM-12162

Table S1 List of the primers used for the creation of the constructs. In the first column is indicated the name of the construct that has been generated; in the second column is indicated the template used for the PCR reactions needed for the creation of the construct; in the third column is indicated the name of the primer used for the PCR reaction; in the last column is indicated the purpose of the reaction in respect to the creation of the final construct. The background color of the cell specifies the cloning strategy that was used: grey is for traditional cloning (sticky-ends ligation), light blue is for site-directed mutagenesis, yellow is for Gibson Assembly. Because of a change in the experimental planning, the cloning of constructs W2 LER2, W2 LER3, and W2 LER4 was obtained by first inserting a 6 glycines-long spacer between the portion of interest and the eGFP, and then by removing the spacer. BB means “backbone”, CDS means “coding sequence”; MCS mean “multiple cloning site”.

Construct	Template	Primers	Use
**pTpNAT-MCS**	plasmid 937	Fcp9promFw	Amplification of NAT cassette
		Fcp9termRv2	
	pPhaNR	pPhaT1MCSfw	Amplification of MCS
		pPhaT1MCSrv	
**Sil3**	plasmid 396<br>pTpNAT-MCS	Sil3EcoRVwholeFw	Insertion of Sil3 gene cassette
		Sil3AccI300DwRv	
**CinW2**	plasmid 936<br>pTpNAT-MCS	CinW2XbaI800upFw	Insertion of CinW2 gene cassette
		CinW2AccIWholeRv	
**CinY2**	plasmid 937	(Gib)CinY2insert_fw	Insertion of CinY2 gene cassette
		(Gib)CinY2insert_rv	
	pTpNAT-MCS	(Gib)Y2insertion_pTpBB_fw	
		(Gib)Y2insertion_pTpBB_rv	
**5357**	gDNA<br>pTpNAT-MCS	5357_SacI800up_fw	Insertion of 5357 gene cassette in pTpNAT-MCS
		5357_XbaI300dw_rv	
	pTp5357	pTp5357_gib_fw	Insertion of eGFP
		pTp5357_gib_rv	
	pTpSil3 (for the eGFP)	CinW2 N-ter_fw	
		eGFP_rv	
**12162**	gDNA	pTp12162_800up(gib)_fw	Insertion of 12162 gene cassette into pTpNAT-MCS
		pTp12162_300dw_rv(gib)CORRECT	
	pTpNAT-MCS	fcp9_p_fw(no r.s.)	
		pTpInvNAT_bb_rv	
	pTpSil3 (for the eGFP)	CinW2 N-ter_fw	Insertion of eGFP into pTp12162
		eGFP_rv(no stop) for 12162(gib)	
	pTp12162	fcp9_fw_12162(gib)	
		pTp12162_300up(gib)_rv	
sil3/cinW2	pTpSil3	(new)D_Sil3_TermFw	Amplification of the Sil3 BB
		D_Sil3_PromRv	
	pTpCinW2	D_CinW2_GeneFw(Sil3_PromFw)	Amplification of the CinW2 CDS
		(new)D_CinW2_GeneRv(Sil3_TermRv)	
sil3/cinY2	pTpSil3	(new)D_Sil3_TermFw	Amplification of the Sil3 BB
		D_Sil3_PromRv	
	pTpCinY2	(new)D_CinY2_GeneFw(Sil3_PromFw)	Amplification of the CinY2 CDS
		(new)D_CinY2_GeneRv(Sil3_TermRv)	
cinW2/sil3	pTpCinW2	(new)D_CinW2_TermFw	Amplification of the CinW2 BB
		D_CinW2_PromRv	
	pTpSil3	D_Sil3_GeneFw(CinW2_PromFw)	Amplification of the Sil3 CDS
		(new)D_Sil3_GeneRv(CinW2_TermRv)	
cinW2/cinY2	pTpCinW2	(new)D_CinW2_TermFw	Amplification of the CinW2 BB
		D_CinW2_PromRv	
	pTpCinY2	(new)D_CinY2_GeneFw(CinW2_PromFw)	Amplification of the CinY2 CDS
		(new)D_CinY2_GeneRv(CinW2_TermRv)	
cinW2/sil3	pTpCinY2	D_CinY2_TermFw	Amplification of the CinY2 BB
		(new)D_CinY2_PromRv	
	pTpSil3	(new)D_Sil3_GeneFw(CinY2_PromFw)	Amplification of the Sil3 CDS
		(new)D_Sil3_GeneRv(CinY2_TermRv)	
cinW2/cinY2	pTpCinY2	D_CinY2_TermFw	Amplification of the CinY2 BB
		(new)D_CinY2_PromRv	
	pTpCinW2	(new)D_CinW2_GeneRv(CinY2_TermRv)	Amplification of the CinW2 CDS
		(new)D_CinW2_GeneFw(CinY2_PromFw)	
**CinW2 1-187**	pTpCinW2	CinW2Trunc_N-ter_fw	Truncation
		CinW2Trunc_N-ter_rv	
**CinW2 188-383**	pTpCinW2	CinW2Trunc_C-ter_fw	Truncation
		CinW2Trunc_C-ter_rv	
**CinY2 1-136**	pTpCinY2	CinY2Trunc_N-ter_fw	Truncation
		CinY2Trunc_N-ter_rv	
**CinY2 137-248**	pTpCinY2	CinY2Trunc_C-ter_fw	Truncation

		CinY2Trunc_C-ter_rv	
**CinW2 MUT-A**	pTpCinW2	W2_PLC1(k-to-r)_fw	Lysine to arginine mutation of W2-LER1
		W2_PLC1(k-to-r)_rv	
**CinW2 MUT-B**	pTpCinW2	CinW2PLC2(K-to-R)fw	Lysine to arginine mutation of W2-LER2
		CinW2PLC(K-to-R)rv	
**CinW2 MUT-C**	CinW2 N-ter PLC1 mut	CinW2PLC2(K-to-R)fw	Lysine to arginine mutation of both LERs in CinW2
		CinW2PLC(K-to-R)rv	
**CinW2 DEL-A**	pTpCinW2	W2_PLC1del_fw2	Deletion of W2-LER1
		W2_PLC1del_rv2	
**CinW2 DEL-B**	pTpCinW2	W2_PLC2del_fw	Deletion of W2-LER2
		W2_PLC2del_rv	
**CinW2 DEL-C**	CinW2 N-ter PLC1 del	W2_PLC2del_fw	Deletion of both LERs from CinW2
		W2_PLC2del_rv	
**W2 LER1**	pTpW2	CinW2_PLC1_fw	Deletion of the region comprised between the SP and the LER1
		CinW2Trunc_C-ter_rv	
	(from previous step)	CinW2Trunc_N-ter_fw	Deletion of the region comprised between the LER1 and the eGFP
		CinW2_PLC1_rv	
**W2 LER2**	pTpW2	W2_SP+GS+GFP_fw	Insertion of a glycine spacer between the LER2 and the eGFP
		W2_SP+GS+GFP_rv	
	(from previous step)	W2_PLC2_fw2	Insertion of LER2
		W2_PLC2_rv2	
	(from previous step)	CinW2Trunc_N-ter_fw	Deletion of the glycine spacer
		W2_PLC2_rv	
**W2 LER3**	pTpW2	W2_SP+GS+GFP_fw	Insertion of a glycine spacer between the LER3 and the eGFP
		W2_SP+GS+GFP_rv	
	(from previous step)	W2_PLC3_fw2	Insertion of LER3
		W2_PLC3_rv2	
	(from previous step)	CinW2Trunc_N-ter_fw	Deletion of the glycine spacer
		W2_PLC3_rv	
**W2 LER4**	pTpW2	W2_SP+GS+GFP_fw	Insertion of a glycine spacer between the PLC and the eGFP
		W2_SP+GS+GFP_rv	
	(from previous step)	W2_PLC4_fw2	Insertion of LER4
		W2_PLC4_rv2	
	(from previous step)	CinW2Trunc_N-ter_fw	Deletion of the glycine spacer
		W2_PLC4_rv	
		Sil3_PLC2_no GS_rv	
**CinW2 1-103**	pTpCinW2 N-ter	CinW2Trunc_N-ter_fw	Truncation
		CinW2_Trunc_N-ter(1-103)rv	
**CinW2 1-103 DEL**	pTpCinW2 N-ter PLC1/PLC2 del	CinW2Trunc_N-ter_fw	Truncation
		CinW2_Trunc_N-ter(1-103)rv	
**CinW2 104-187**	pTpCinW2 N-ter	CinW2_Trunc_N-ter(104-187)fw	Truncation
		CinW2Trunc_C-ter_rv	
**CinW2 104-187 DEL**	pTpCinW2 N-ter PLC1/PLC2 del	W2-N-ter-PLC1/2del(104-187)_fw	Truncation
		CinW2Trunc_C-ter_rv	
**CinY2 1-72**	pTpCinY2 N-ter	CinY2Trunc_N-ter_fw	Truncation
		CinY2_Trunc_N-ter(1-72)rv	
**CinY2 73-136**	pTpCinY2 N-ter	CinY2_Trunc_N-ter(73-136)fw	Truncation
		CinY2Trunc_C-ter_rv	
**CinY2 UM**	pTpCinY2	CinY2Trunc_N-ter_fw	Truncation
		CinY2_17aa_afterSPrv	
**CinY2 UM DEL**	pTpCinY2	Y2_17aa del_fw	Truncation
		CinY2Trunc_C-ter_rv	
		Sil3_SP_rv	
**CinW2 SP**	pTpCinW2	CinW2Trunc_N-ter_fw	Truncation
		CinW2Trunc_C-ter_rv	
**CinY2 SP**	pTpCinY2	CinW2Trunc_N-ter_fw	Truncation
		CinY2Trunc_C-ter_rv	
		Sil3_SP_rv	

Table S2 Sequences of the primers listed in Table S1 (the primers are listed in alphabetical order).

PRIMER NAME	PRIMER SEQUENCE (5'-3')
(Gib)CinY2insert_fw	GGTACCCGGGGATCCTCTAGAGGTGGTGGTGTTGGCTGCTAGC
(Gib)CinY2insert_rv	GCTTGCATGCCTGCAGGTCGACCTAACAAATACTAATTCGCTGTTGC
(Gib)Y2insertion_pTpBB_fw	GTCGACCTGCAGGCATGCAAGC
(Gib)Y2insertion_pTpBB_rv	TCTAGAGGATCCCCGGGTACC
(new)D_CinW2_GeneFw(CinY2_PromFw)	ATCATACAGCAAATCGACAATATATACAATGAAGCTCGCTCTCTTCCTAACGATCC
(new)D_CinW2_GeneRv(CinY2_TermRv)	CGGCATGGACGAGCTGTACAAGTAAGTGTTGATGTCTCCTTCAAGTGACACC
(new)D_CinW2_GeneRv(Sil3_TermRv)	CGGCATGGACGAGCTGTACAAGTAAGTTCATCATCTTCATATCGTATGAAGTGG
(new)D_CinW2_TermFw	TAAATAACCACAACTATCTACC
(new)D_CinY2_GeneFw(CinW2_PromFw)	CTCAACGATAAACGAACAAAAGAAACCATGAAGTTAATCATCGCCCTCAGCGC
(new)D_CinY2_GeneFw(Sil3_PromFw)	CCTATCAACACATCACCATATCAAAAATGAAGTTAATCATCGCCCTCAGCGC
(new)D_CinY2_GeneRv(CinW2_TermRv)	GGTGCTTCTCACAACAACAGGATATAATAAATAACCACAACTATCTACC
(new)D_CinY2_GeneRv(Sil3_TermRv)	GGTGCTTCTCACAACAACAGGATATAAGTTCATCATCTTCATATCGTATGAAGTGG
(new)D_CinY2_PromRv	ATCATACAGCAAATCGACAATATATACA
(new)D_Sil3_GeneFw(CinY2_PromFw)	ATCATACAGCAAATCGACAATATATACAATGAAGACTTCTGCCATTGTATTGC
(new)D_Sil3_GeneRv(CinW2_TermRv)	GGACGAGCTGTACAAGTAATAAATAACCACAACTATCTACC
(new)D_Sil3_GeneRv(CinY2_TermRv)	GGACGAGCTGTACAAGTAAGTGTTGATGTCTCCTTCAAGTGACACC
(new)D_Sil3_TermFw	GTTCATCATCTTCATATCGTATGAAGTGG
5357_SacI800up_fW	GAGCTCCCTTTGGATTCAACG
5357_XbaI300dw_rv	TCTAGACTTTCTGGAAGTTTGACG
CinW2 N-ter_fw	ATGGTGAGCAAGGGCGAGGAGC
CinW2_PLC1_fw	AAATCTGGCAAGTCCGGATCGG
CinW2_PLC1_rv	CTTGCCGGACTTGCCCGATCC
CinW2_Trunc_N-ter(1-103)rv	GGAGTCATCTTCAATGTACACC
CinW2_Trunc_N-ter(104-187)fw	AGCGATGGCAGTGGTAAGAGTGG
CinW2AccIWholeRv	GTCGACCTACATCAAATGAGATGAAG
CinW2PLC(K-to-R)rv	GGAAGACGATCCCCTGCCACTCCTACCACTGCCATCGCTGGA
CinW2PLC2(K-to-R)fw	AGGGGAAGCAGGGGGAGTAGCAGGAGTAGTAGGGGAAGCAGTAGTGATG
CinW2Trunc_C-ter_fw	GATGATTCCTCTTCCTCCAAGTCAAGCAAGGG
CinW2Trunc_C-ter_rv	GGCGGCAATGAGGGTGGGGATCG
CinW2Trunc_N-ter_fw	ATGGTGAGCAAGGGCGAGGAGC
CinW2Trunc_N-ter_rv	ATCGTCAGATCCCCAGTGCGAGG
CinW2XbaI800upFw	TCTAGATTGCACAGACCTAACTAATGGTGTC
CinY2_17aa_afterSPrv	GGGGGTAGGACGGCCTGGGAATGG
CinY2_Trunc_N-ter(1-72)rv	CAAGGTAGGAAGAGGAGTAGGC
CinY2_Trunc_N-ter(73-136)fw	GGGGGGGTTCCTACGACGAAGATGC
CinY2Trunc_C-ter_fw	GACAACTACGCCGACGACTACACC
CinY2Trunc_C-ter_rv	TGCCGAAGCGAGGGTGATTGC
CinY2Trunc_N-ter_fw	ATGGTGAGCAAGGGCGAGGAGC
CinY2Trunc_N-ter_rv	TCCGTATCCGTATCCTGTTGTTAGATGTTGGAGG
D_CinW2_GeneFw(Sil3_PromFw)	CCTATCAACACATCACCATATCAAAAATGAAGCTCGCTCTCTTCCTAACGATCC
D_CinW2_PromRv	CTCAACGATAAACGAACAAAAGAAACC
D_CinY2_TermFw	GTGTTGATGTCTCCTTCAAGTGACACC
D_Sil3_GeneFw(CinW2_PromFw)	CTCAACGATAAACGAACAAAAGAAACCATGAAGACTTCTGCCATTGTATTGC
D_Sil3_PromRv	CCTATCAACACATCACCATATCAAAA
eGFP_rv	TTACTTGTACAGCTCGTCCATGCCG
eGFP_rv(no stop) for 12162(gib)	CTACTGACGCAACATCCTCTTGTACAGCTCGTCCATGCCG
fcp9_fw_12162(gib)	AGGATGTTGCGTCAGTAGATTAGTTATGCGCCGCTCTTTCTCG
fcp9_p_fw(no r.s.)	GCAACGAATATTTCCAGAAGG
Fcp9promFw	TCCGGAGCAACGAATATTTCCAGAAGGAG
Fcp9termRv2	CTCGAGTAATGGATCTAGGGGATGCT
pPhaT1MCSfw	CATATGGAATTCGATATCATCGACTA
pPhaT1MCSrv	TCCGGAAAGCTTGCATGCCTGCAGG
pTp12162_300dw_rv(gib)CORRECT	TGGAAATATTCGTTGCCAATACCAGTCGTTTTATATGG
pTp12162_300up(gib)_rv	CGCCCTTGCTCACCATCCTTCCCTTTCCCGACTTTCC
pTp12162_800up(gib)_fw	AGATTGTACTGAGAGTGCACTTTCGTACTGTATGAGCGTCG
pTp5357_gib_fw	TGGACGAGCTGTACAAGTAAAGGGTGCAAATACTGATTCATGAATTG
pTp5357_gib_rv	TCCTCGCCCTTGCTCACCATTGGCACTGCTGCCAAAGTC
pTpInvNAT_bb_rv	GTGCACTCTCAGTACAATCTGC
Sil3_PLC2_no GS_rv	GAAGATCTTGGCAGCCTTGGCAGCAGTGGTGGCGAGAACG
Sil3_SP_rv	GGCAGCAGTGGTGGCGAGAACG
Sil3AccI300DwRv	GTCGACCCTCACTGATCCATACGATG
Sil3EcoRVwholeFw	GATATCCGGCAGGATACTGATGATAG
W2_PLC1(k-to-r)_fw	TCGGGCAGGTCCGGCAGGGGAAGTAGTGG
W2_PLC1(k-to-r)_rv	TCCGGACCTGCCAGATCTGGAACTGG
W2_PLC1del_fw2	GGAAGTAGTGGACCACACGGC
W2_PLC1del_rv2	GGAACTGGAGTAGTCTCCTCCCCATC
W2_PLC2_fw2	AAGCAAGGGGAGTAGCAAGAGTAGTAAGGGAAGCAGTGGTGGCGGAGGGATG GTGAGC

W2_PLC2_rv	ACTGCTTCCCTTACTACTCTTGCTACTCC
W2_PLC2_rv2	CCCTTGGAAGACGATCCCTTGCCACTCTTACCACTGCCGGCGGCAATGAGGGT GGGGATCG
W2_PLC2del_fw	GGAAGCAGTAGTGATGATAGTACGG
W2_PLC2del_rv	ACCACTGCCATCGCTGGAG
W2_PLC3_fw2	CGTCCAAGTCAAGCAAGGGATCGTCGGGTGGCGGAGGGATGGTGAGC
W2_PLC3_rv	CGACGATCCCTTGCTTGACTTGG
W2_PLC3_rv2	ATCCCTTGGAAGACTTGCTGCTTGAGGCGGCAATGAGGGTGGGGATCG
W2_PLC4_fw2	TCGTCCAAGTCATCCAAGGGTTCATCCGGTGGCGGAGGGATGGTGAGC
W2_PLC4_rv	GGATGAACCCTTGGATGACTTGG
W2_PLC4_rv2	TGATCCCTTGCTTGACTTGGATGAGTCGGCGGCAATGAGGGTGGGGATCG
W2_SP+GS+GFP_fw	GGAGGGATGGTGAGCAAGGGCGAGG
W2_SP+GS+GFP_rv	GCCACCGGCGGCAATGAGGGTGGGG
W2-N-ter-PLC1/2del(104-187)_fw	AGCGATGGCAGTGGTGGAAGC
Y2_17aa del_fw	AACCCCACAATGGTGAATACCATCG
